# Stress Fracture of the Proximal Fibula in Military Recruits

**DOI:** 10.4055/cios.2009.1.3.161

**Published:** 2009-08-17

**Authors:** Seoung Hwan Hong, In Tak Chu

**Affiliations:** Department of Orthopaedic Surgery, Seoul St. Mary's Hospital, The Catholic University of Korea, School of Medicine, Seoul, Korea.

**Keywords:** Proximal Fibula, Stress fracture, Military recruits

## Abstract

**Background:**

We wanted to report on stress fracture of the proximal fibula and to suggest the pathomechanism of this fracture.

**Methods:**

Between April 2004 through April 2005, the military recruits who complained of leg pain during the 6 weeks basic training in the Republic of Korea Marine Corps education and training group were evaluated according to their clinical manifestations and plain radiographs.

**Results:**

Twelve recruits of 635 recruits who complained leg pain were diagnosed as having fibular stress fracture. Eleven cases (10 recruits) appeared at the junction of the proximal and middle 1/3 of the fibula and 2 cases (2 recruits) were in the middle 1/3 of the fibula, as assessed radiologically. Tenderness was the most reliable clinical manifestation. All the fractures occurred after repetitive walking or jumping in a squatting position. Conservative treatments that included bed rest, immobilization and non-steroidal anti-inflammatory drugs administration according to the symptom severity were satisfactory.

**Conclusions:**

Proximal fibular stress fracture is not rare in military recruits. The shearing force on the proximal fibula and the repetitive stress by walking or jumping in a squatting position contribute to the stress fracture of the proximal fibula.

Stress fractures are generally defined as overuse injuries that are attributable to the repetitive loading of bone with performing vigorous weight bearing activity such as running, jogging and marching.1) The symptoms of stress fractures are characterized as dull localized swelling and pain of an insidious onset, and these symptoms are aggravated by physical activity and they are relieved by rest. There are two types of stress fracture.^2^,3) A fatigue fracture that occurs when repeated abnormal stress is applied to normal bone, and a insufficiency fracture that occurs when normal stress is applied to abnormal bone. Increased bone stress may cause local weakening of the bone due to the unbalancing osteoclastic activity, which leads to microfracture.4)

Although stress fractures may be seen in almost every bone in the human body, the most frequent sites in athletes are the tibia shaft followed by metatarsals, femur and distal fibula. Proximal fibular stress fracture is extremely rare and only few case reports on athletes and military populations have been published.^5^-9)

The exact etiology of proximal fibular stress fracture is unknown and there had been no report concerned with the pathomechanism of stress fracture of the proximal fibula. The purpose of this retrospective cohort study is to determine the pathomechanism of stress fracture of the proximal fibula and to document the results of conservative treatment.

## METHODS

Between April 2004 through April 2005, we evaluated 12 military recruits (13 cases) who were in 6 weeks of basic training in the Republic of Korea Marine Corps education and training group. The basic training consisted of increasing the level of muscle strength via exercise such as running, marching and mountain climbing. All the subjects were men and their mean age was 21.3 years (range, 20 to 22 years). They are all physically healthy and they received training 8 hours a day. Six of the 12 recruits smoke cigarettes, yet none had a history of steroid use, foot deformity or rheumatoid ar thritis. They visited our clinic at an average of 10.8 days (range, 1 day to 1 month) after walking or jumping in a squatting position, which was a part of the physical exercises for strengthening the quadriceps femoris. The diagnoses were made by physical examination and according to the plain radiographic findings. All cases were treated by conservative methods that include immobilization by a short leg splint and non-steroidal anti-inflammatory drugs (NSAIDs), if needed. Follow-up radiographs were taken every 2 weeks and the patients were allowed to return to training when the symptoms had subsided.

## RESULTS

**Fig. 1:**
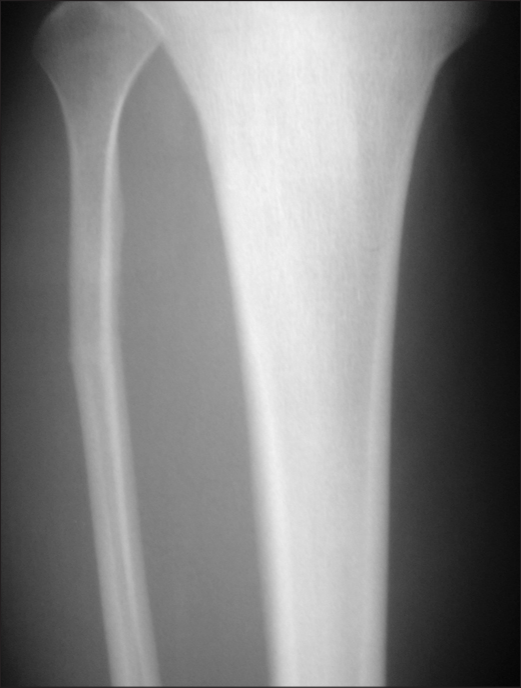
A plain tibial anteroposterior radiograph of a 21 year-old man shows a proximal fibular stress fracture with some lateral angulation.

**Fig. 2:**
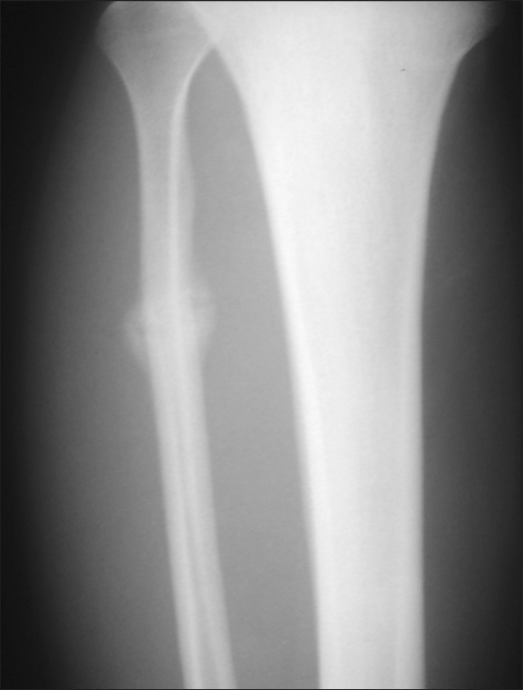
Two weeks after symptom onset, a callus was formed at the fracture site.

All the patients complained of proximal leg pain, which was aggravated by the squatting position. Physical examinations revealed swelling and characteristic severe tenderness at the superolateral aspect of the leg. Radiographically, the fracture line occurred at the junction of the proximal and middle 1/3 of the fibula in 11 cases (10 recruits) and at the middle 1/3 in 2 cases (2 recruits). On the anteroposterior view, the fracture sites were displaced 0.8 mm (range, 0 to 2 mm) on average and angulated laterally 6.8° (range, 0 to 10°) on average (Fig. 1). All the cases revealed a subperiosteal reaction by the callus formation at about 2 weeks after symptom onset (Fig. 2), and the reaction mimicked osteomyelitis or osteosarcoma; however, no case showed osteolytic evidence of the cortex.

The treatments consisted of bed rest, immobilization and NSAIDs according to the severity of the symptoms. Most of the patients were prescribed 7 days bed rest and immobilization, and then they started active ankle motion exercise and partial weight bearing with crutches. Full weight bearing was restored at 14 days. There was no impediment of ambulation, but running or strenuous activity was impossible. The tenderness gradually subsided 2 weeks after symptom onset, and the callus was formed. Then all the training except walking or jumping in a squatting position could be performed.

The tenderness disappeared and the patients were able to perform all the training activities at 4 weeks after the onset of the initial symptoms. No case showed refracture.

## DISCUSSION

Stress fractures were first described by a Prussian army doctor Breithaupt in 1855,10) and it is a syndrome that's characterized by edematous and painful feet following long marches. Since then, stress fractures have been rec ognized as a common injury of military recruits. Recently, not only military recruits but athletes too are prone to stress injury.^11^-13) Several risk factors for bone stress fracture have been described and categorized into the nonmodifiable and modifiable risk factors.1) The nonmodifiable risk factors include the female gender, Caucasian ethnicity and some diseases that enhance bone turnover.^14^,15) The modifiable risk factors include poor physical fitness, smoking,15) steroid use, low levels of sex hormones, low bone density and footwear.14)

None of the cases in our study had risk factor according to the routine hematologic and radiologic examinations that were performed before admission to the military.

There are two mechanisms of stress injury. One theory is that muscle fatigue from prolonged exertion allows more force to be transmitted to the relatively less protected bone, and the other is that highly concentrated muscle forces act across the normal bone in a repetitive and attritional fashion.16) Our cases were young, active man who had normal bone quality, and strong contractile muscle force may have acted to create a fatigue fracture.

The diagnosis of stress fracture is primarily a clinical one.5) The clinical features are localized tenderness and pain on exertion. Simple radiologic studies and a bone scane are useful to make an early diagnosis.7) During the acute period, a pathologic examination is generally not warranted for this type of fracture since the findings may be mistaken for osteosarcoma.16) In a previous study, the normal signal intensity on MRI from the bone marrow was the major evidence against osteomyelitis or neoplasm, and this illustrated the usefulness of MRI in the chronic stage.17) MRI is believed to be more specific for making the diagnosis of stress fracture than bone scanning.6) Particularly, a thorough history taking, physical examination and plain radiographic follow-up are the keys for diagnosing and assessing the acute stage, and MRI is seldom needed for examining patients in this stage.

The common sites of stress fractures are tibia, metatarsal, femur and fibula.^11^,^12^,18) Biomechanical studies have investigated the role of the fibula for weight bearing and they have found that the fibula carries between 6.4% and 16.7% of the load applied to the lower extremity.6) For the cases of fibular stress fracture, the most common site is the distal 1/3.^19^,20) A proximal 1/3 fibular stress frac ture has rarely been reported^5^-8) and jumping seems to have an important role in the genesis of a high fibular stress fracture.7) However, the etiology of proximal stress fracture remains unclear, yet Blair and Hanley21) suggested a combination of compression loading, the stresses of biceps femoris contraction and shear fatigue.

The gastrocnemius muscle originates from the distal femur and the soleus muscle originates from the proximal tibia and fibula. Ankle plantar flexion for jumping and walking in a squatting position require triceps surae muscle contraction, yet the gastrocnemius muscle works less as compared to the soleus muscle because the knee joint is maximally flexed in a squatting position. Jumping in a squatting position requires soleus muscle contraction. Walking in a squatting position requires contraction of the ankle plantar flexor (soleus) for heel-off and ankle evertor (peoneus brevis) for toe-off. The peroneus brevis originates at the inferior 2/3 of the lateral aspect of the fibula.

Our cases revealed stress fracture at the junction of the proximal and middle 1/3 of the fibula in 11 (84%) of 13 cases. So the alternating muscle contraction of the soleus and peroneus brevis muscle during walking rather than during jumping in a squatting position may act to place shearing force on the proximal fibula and the repetitive stress contributes to the fracture of the proximal fibula.

Proximal fibular stress fracture is not rare in military recruits. The shearing force placed on the proximal fibula and the repetitive stress due to walking or jumping in a squatting position contributes to the stress fracture of the proximal fibula.
